# Microbial Signatures of Obesity-Aggravated Psoriasis: Insights from an Imiquimod-Based Mouse Model

**DOI:** 10.3390/ijms26167697

**Published:** 2025-08-08

**Authors:** Carolina Constantin, Elena-Georgiana Dobre, Paula Istvan, Adriana Narcisa Munteanu, Mihaela Surcel, Gheorghita Isvoranu, Monica Neagu

**Affiliations:** 1 Immunology Department, “Victor Babes” National Institute of Pathology, 050096 Bucharest, Romania; caroconstantin@gmail.com (C.C.); iadinuta@yahoo.com (A.N.M.); msurcel2002@yahoo.com (M.S.); neagu.monica@gmail.com (M.N.); 2 Colentina Clinical Hospital, 020125 Bucharest, Romania; 3 European Virus Bioinformatics Center, University of Jena, 07743 Jena, Germany; 4 Animal Husbandry, “Victor Babes” National Institute of Pathology, 050096 Bucharest, Romania; gina_isvoranu@yahoo.com; 5 Faculty of Biology, University of Bucharest, 050095 Bucharest, Romania

**Keywords:** psoriasis, microbiota, dysbiosis, diet, 16S rRNA sequencing

## Abstract

As obesity and Western diet consumption are key factors contributing to gut dysbiosis, we investigated the relationship between intestinal microbiota, obesity, and psoriasis in an imiquimod-based model. C57BL/6 mice were used as follows: psoriasis-induced groups fed continuously with a standard or Western diet, psoriasis-induced group fed with a Western diet and then returned to a standard diet, and controls. For each group, clinicopathological, immune, and metabolic parameters were integrated with microbiome data. The imiquimod-based models displayed human psoriasis features and significant changes in immune parameters. Hence, psoriatic mice on prolonged high-fat intake presented decreased microbial richness and evenness and a gut microbiome composition resembling that of obese mice. *Ruminococcus*, *Clostridium*, *Desulfovibrio*, and *Enterorhabdus* were the most abundant genera in the obesity-enhanced psoriasis group. *Raoultella* abundance was linked with psoriasis. Yet, the same pathobionts over-represented in the obese psoriatic mice displayed positive correlations with metabolic stress indicators and proinflammatory factors, indicating potential biomarkers of disease severity. Conversely, *Lactobacillus taiwanensis*, *Alistipes putredinis*, and *Eubacterium hadrum* might be potential taxa for attenuating the metabolic burden in obesity-enhanced psoriasis. Here, we depict the microbial signatures associated with inflammation and metabolic stress in an obesity-aggravated psoriasis mouse model.

## 1. Introduction

Psoriasis (Pso) is an autoimmune, chronic, recurrent dermatitis. Accumulating data show a link between gut microbiota dysbiosis and an exacerbated Pso condition. Moreover, as obesity and Western diet consumption may trigger dysbiosis of gut microbiota [1], we intend to investigate the relationship between intestinal microbiota, diet-induced obesity, and Pso development in an established mouse model.

Skin and gut intimate contact play an important role in the development of Pso. For instance, increased interleukin (IL)-17A in the gut leads to systemic IL-17, affecting skin inflammation [2]. Normal intestinal microbiota (e.g., phyla *Firmicutes*, *Bacteroidetes*, *Actinobacteria*, *Proteobacteria*, and *Verrucomicrobia*) compete and inhibit the growth of pathogenic microorganisms. Pathological and physiological changes, like aging, induce alterations in the intestinal microbiota, reductions in *Bacteroidetes* (e.g., *Bacteroides*) and *Actinobacteria* (e.g., *Bifidobacterium*), and an increase in pathogenic *Firmicutes* (e.g., *Clostridium*) [3]. Moreover, intestinal microbiota is actively involved in the regulation and maturation of the immune system. For example, *Akkermansia muciniphila* generates the beneficial short-chain fatty acids (SCFAs) acetate, propionate, and butyrate, promotes the integrity of the mucosal barrier as it impedes pathological species [4] through antibiotic peptides [5], and stimulates the maturation of regulatory lymphocytes [6]. In Pso, intestinal *Akkermansia* and *Ruminococcus* decrease [7] and do not regulate T regulatory (Treg) cell differentiation any longer, resulting in altered immune responses [8]. IL-17 is an important player in inflammation, mediating metabolic syndrome and Pso [9,10,11]. Besides IL-17 (IL-17A/IL-17F), Th17 cells produce an array of cytokines, like tumour necrosis factor (TNF)-α and IL-22 [12], and all these contribute to an altered differentiation and hyperproliferation of keratinocytes. In patients diagnosed with Pso, even bacterial DNA was found in circulation, enhancing the systemic inflammatory response [13,14]. In general, during Pso development, authors agree on a loss of microbial diversity [15]. An important experiment performed on an imiquimod (IMQ)-induced model of skin inflammation showed that *Staphylococcus aureus* and *Streptococcus danieliae* administered orally increased the psoriatic skin lesions and further increased TNF-α, IL-17, and IL-22 cytokine levels [16]. Reduced skin inflammation was also found to be correlated with low Th17 cells residing in the spleen and lymph nodes [17].

Recent studies have shown that more than 50% of Pso patients have an increased density of intestinal eosinophils, mast cells, macrophages, and T-CD8^+^ cells that express activation markers correlated with increased mucosal IL-17A, IL-13, IL-2, and IL-20 expression [18]. This entire array of participating immune cells is jointly contributing to the elevated levels of cytokines associated with increased intestinal permeability. Besides Th17-type CD4, IL-17A is produced by various cells, such as inflammatory cells, neutrophils, mast cells, eosinophils [19,20], γδ T cells [21], group 3 innate lymphoid cells (ILC3) [22], and mucosal-associated invariant T (MAIT) cells [23], IL-20 is produced by monocyte-derived macrophages [24], and IL-13 is produced by T-CD8^+^ cells in lesional psoriatic skin [25].

The severity of Pso lesions has been studied in recent years in correlation with obesity, as the metabolic syndrome can be an important deregulating factor of gut microbiota. Hence, in a meta-analysis with over 2 million individuals, in which 1/10 were psoriatic patients, the risk of obesity exceeded 50% compared to healthy subjects. This risk increased with the form of Pso, and normal-weight patients were found to become obese in the future [26]. Adipose tissue contributes to the inflammatory milieu and maintains chronic low-grade systemic inflammation [27,28]. An altered gut microbiota can be a significant trigger in obesity development [29], and microorganisms that inhabit the internal gastrointestinal system are involved in autoimmune diseases [30]. *Firmicutes*, *Bacteroides*, *Proteobacteria*, and *Actinobacteria* make up more than 98% of the gut microbiota. In Pso, studies are accumulating that demonstrate the lowering of abundances of *Proteobacteria* and *Bacteroides*, while the abundances of *Actinobacteria* and *Firmicutes* increase [31,32]. Like Pso, in obese individuals, significant alterations of the gut microbiota are reported where the *Firmicutes* and *Bacteroidota* (F/B) ratio is altered compared to normal-weight subjects. The F/B ratio is recognized as a marker of normal gut homeostasis [31]. Variations in the F/B ratio induce lead dysbiosis of various grades, and are associated with obesity and various inflammatory conditions, including Pso [33,34,35]. The obese psoriatic patient is also characterized by chronic pro-inflammatory cytokines and adipokines that are generated by white fat adipocytes; high levels of IL-17, IL-23, TNF, and interferon (IFN) were reported to support the gut microbiome–obesity relationship in Pso patients [36,37,38,39].

IMQ-induced psoriatic mice were the first established model and have become the most used animal model of Pso. IMQ is an agonist of toll-like receptor 7 (TLR-7), inducing skin inflammation, and the inflicted skin condition has human Pso characteristics [40]. Using this type of model, important evidence has been found concerning the skin–gut axis. Several years ago, Zanvit et al. reported in this animal model that depletion of the microbiota through antibiotics ameliorated Pso skin inflammation [41], because the microbiota was found to be disturbed by Pso induction [42]. *Lactobacillus* was the predominant genus in the gut microbiota of psoriatic mice treated with antibiotics [43]. Although there are still contradictory results regarding the skin microbiota in the Pso animal model, significant deregulations were reported in the alpha- and beta-types of bacteria comprising the skin’s microbiota. In the IMQ-induced Pso experimental model, the gut microbiota comprises increased abundances of *Lactobacillus* (*L. intestinalis*, *L. reuteri*, and *L. taiwanensis*), *Bacteroides*, and *Staphylococcus* species [44]. Yet, a marked reduction in the *Parabacteroides*, *Faecalimonas*, and *Alistipes* genera was noted in the IMQ-induced Pso mouse models compared to the controls. Several years ago, it was demonstrated that intestinal microbiota promotes IMQ-induced skin inflammation and furthermore augments the Th17 response [17]. In another animal model of IMQ-induced Pso, stool samples harvested from healthy donors and concomitantly from psoriatic patients were used to populate mice’s gut flora. Interestingly, the evolution of Pso in the IMQ-based model was intensified when a psoriatic patient’s stool was transplanted in comparison to the healthy donor stool. Healthy microbiota rich in *Lactobacillus reuteri* induced protection against the Treg/Th17 imbalance [45].

As obesity and Western diet consumption play crucial roles in the development of gut dysbiosis, we focused on exploring the relationship between intestinal microbiota, diet-induced obesity, and Pso development in an established mouse model. Therefore, in this study, we aimed to identify potential microbial changes linked to obesity-enhanced Pso in a murine experimental model to uncover novel microbiome-targeted strategies that may mitigate Pso severity.

## 2. Results

### 2.1. Dietary Induced Obesity in Psoriatic Mouse Model

**Figure 1:**
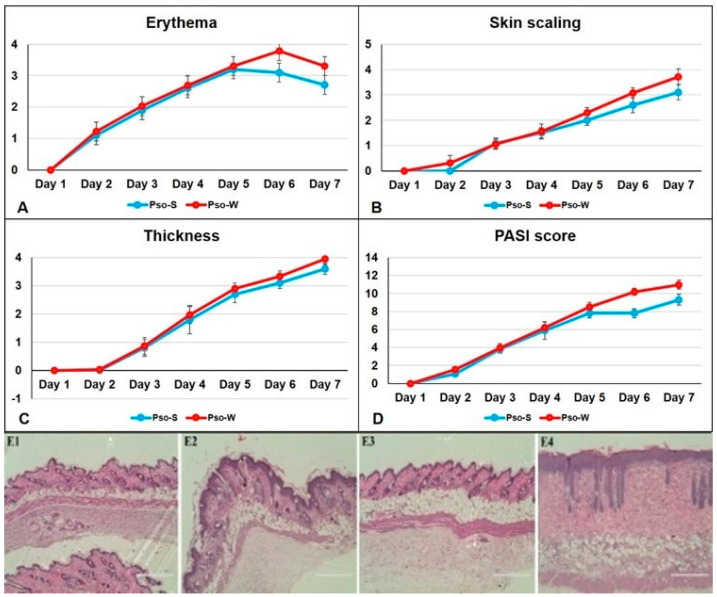
Comparative evolution of skin inflammatory parameters in Pso-W and Pso-S groups. Daily scoring for erythema (**A**), skin scaling (**B**), thickness (**C**), and PASI score (**D**) for Pso-W (*n* = 18) and Pso-S (*n* = 8) groups; data are presented as mean score ± SD (see Supplementary Material Table S1); *n* = number of mice. (**E**) H&E staining of the back skin samples provided from Ctrl-S (**E1**), Pso-S (**E2**), Ctrl-W (**E3**), and Pso-W (**E4**) groups (scale bar = 400 μm).

The mouse psoriatic model is an induced IMQ model of acute psoriasiform alteration of the skin developed by several groups, including ours [46]. It follows the acute phase of human skin Pso and is characterized and hence evaluated by the same parameters as patients. The severity of dorsal skin lesions was evaluated using the PASI score, and clinical observations were conducted daily. For both experimental groups, mice were fed the S and W diets, and the assessment of skin inflammation induced by IMQ-based cream was performed via daily scoring of inflammatory parameters: erythema, skin scaling, and thickness. By summing these parameters, a modified PASI score was calculated daily. Thus, the parameters that comprise the PASI score for Pso evaluation in our animal model are presented in Figure 1. Figure 1D clearly shows that the PASI scores in animals fed the W diet are higher compared to the S diet, and the PASI score is statistically different beginning on day 5 of the Pso installation (Table 1). Overall skin inflammation is increased in the W group. The histopathology of the induced Pso (Figure 1, panels E2 and E4) has all the typical human features for psoriatic lesions: hyperkeratosis, parakeratosis, acanthosis, and elongation of the red ridges. None of these characteristics were observed in healthy mice (control groups) (Figure 1, panels E1 and E3). Moreover, subcutaneous fat can be seen in the Pso-W group, where the inflammatory pattern of the skin is enhanced by the subcutaneous fat deposition.

1. Body weight evolution

**Figure 2:**
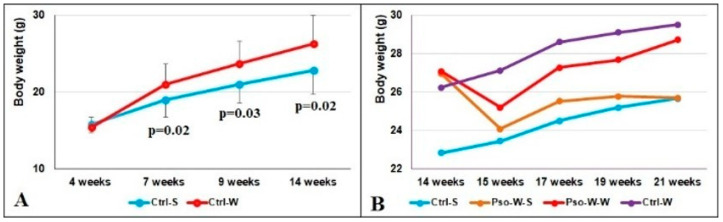
Evolution of body weight in experimental groups. (**A**). Body weight evolution in Ctrl-S and Ctrl-W groups until 14 weeks of age; the results are presented as mean body weight ± SD. (**B**). Body weight evolution in Ctrl-S, Ctrl-W, Pso-W-S, and Pso-W-W groups until 21 weeks of age; the results are presented as mean values for body weight (see Supplementary Material Table S2).

Weighing the mice weekly allowed us to observe that after 3 weeks of the W diet (7 weeks of age), the weights of the mice increased significantly. Statistically significant differences were obtained between the Ctrl-W and Ctrl-S groups and are presented in Figure 2A (*p* < 0.05 at 7, 9, and 14 weeks of age). Interestingly, immediately after Pso onset, the animals’ weights plunged in week 15 (Pso induction is associated with weight loss), but they regained the increment in the following weeks (Figure 2B). The highest weight increment was registered in mice fed the W diet (Ctrl-W group), which was even higher than the Pso-W-W group.

2. Splenomegaly assessment

**Figure 3:**
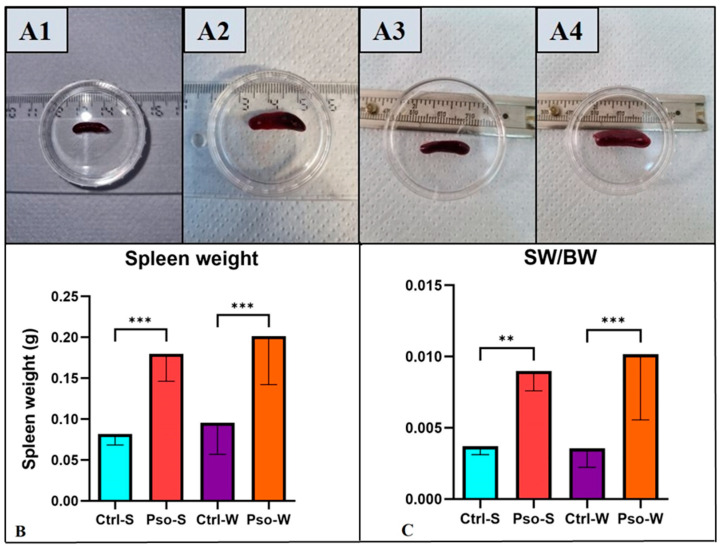
Splenomegaly assessment. (**A**) Representative images of spleen harvested from Ctrl-S (**A1**), Pso-S (**A2**), Ctrl-W (**A3**), and Pso-W (**A4**) mice; (**B**) weights of the spleens in Ctrl-S vs. Pso-S groups and Ctrl-W vs. Pso-W groups; (**C**) the SW/BW ratio in Ctrl-S vs. Pso-S groups and Ctrl-W vs. Pso-W groups; the results are presented as mean spleen weight ± SD and mean ratio SW/BW ± SD (see Supplementary Material Table S3). ** *p* < 0.01; *** *p* < 0.001.

To investigate if our Pso model has secondary immune organ involvement, we weighed the spleens. Figure 3A shows that the Pso groups (fed both S and W diets) had the same weight statistically but more than double compared to controls subjected to the S or W diet. The same assertion is also verified for the ratio between spleen and body weight, proving that the immune response is triggered more by Pso induction and less by the diet (Figure 3B,C).

3. Cholesterol and triglycerides

To investigate if the dietary regimen induced systemic alterations, we investigated total cholesterol (TC) and triglyceride (TG) levels compared to control mice. TC analysis revealed significantly increased values for the groups of mice fed the W diet. Thus, statistically significant differences (*p* ≤ 0.0001) were obtained between the control groups (Ctrl-S vs. Ctrl-W), as well as between the groups of mice with induced Pso (Pso-S vs. Pso-W) (see Supplementary Material Table S4). These data are in agreement with the experimental model of diet-induced obesity published in 2022 [47], where mice received a high-fat diet for 3 months. Sun et al. reported that obese mice had higher serum levels of TC, high-density lipoprotein cholesterol, and low-density lipoprotein cholesterol and lower TG than control mice. In contrast, our data regarding TG values showed only comparable values for all experimental groups [47]. A possible explanation could be that although the fattening period was similar, we used a W diet that is lower in calories than the one reported by Sun et al. [47].

### 2.2. Immune Cell Populations in Dietary Induced Obesity Augmenting Pso

**Figure 4:**
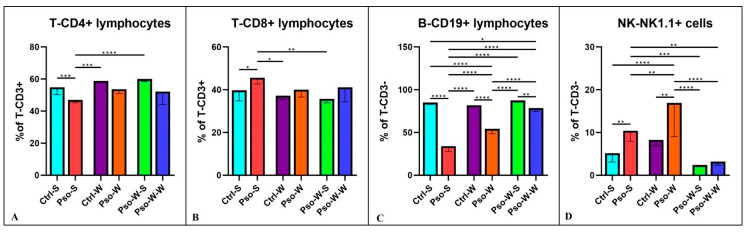
Lymphocytes’ distribution in peripheral blood. Percentage distribution of T-CD4^+^ (**A**), T-CD8^+^ (**B**), B-CD19^+^ (**C**), and NK-NK1.1^+^ (**D**) cells in all experimental groups; the results are presented as percentages of T-CD3ε^+^ and T-CD3ε^−^ lymphocytes (mean ± SD) (see Supplementary Material Table S5). * *p* < 0.05; ** *p* < 0.01; *** *p* < 0.001; **** *p* < 0.0001.

To investigate the profile of immune cells that can sustain systemic inflammation, we tested several immune cell populations. Figure 4 shows that the percentages of T-CD4^+^ are decreased compared to the control in all the psoriatic settings, whether fed the S or W diet, and a significant statistical difference was only observed between Pso-S and Ctrl-S groups (*p* ≤ 0.001). Our data show that the helper T lymphocyte sub-population is affected by the psoriatic event and not by the diet (Figure 4A). When we analyzed the T-CD8^+^ sub-population, the Pso groups had higher percentages of this circulating T subset compared to controls, and the difference was only statistically significant for the Pso-S and Ctrl-S comparison (Figure 4B). As published by us [48] and other groups [25], T-CD8^+^ cells are the main source of IFN-γ, IL-13, IL-17, and IL-22. Another interesting finding previously published by us and confirmed again in this model is the circulatory pattern of B lymphocytes and NK cells (Figure 4C,D). In the Pso groups, the statistically low level of circulatory B cells is doubled by the high levels of NK cells. It is a compensatory phenomenon identified in various autoimmune diseases [49,50], other skin diseases [51,52], and even in respiratory infections [53]. It is highly probable that the imbalance in B-NK that we are witnessing in this model can be explained by the increase in an inflammatory immune population, namely NK-B cells [54], which are switching from strict CD19^+^ B cells to double-positive NK1.1.-CD19 lymphocytes, hence increasing the identified NK cells in the circulation of psoriatic mice.

For all lymphocyte subsets, normalization of values can be observed after administration of the S diet, and practically no statistically significant differences were obtained between the Ctrl-S and Pso-W-S groups.

### 2.3. Circulatory Cytokines/Chemokines Increasing the Inflammatory Status of Diet-Induced Obesity in Pso

**Figure 5:**
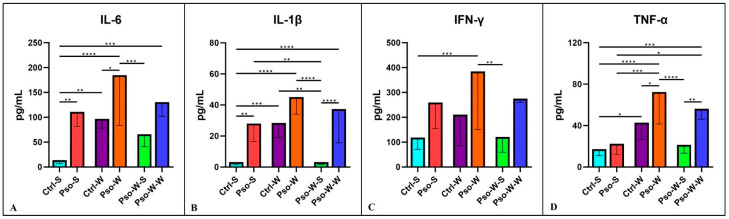
Serum levels distribution of IL-6 (**A**), IL-1β (**B**), IFN-γ (**C**), and TNF-α (**D**) in all experimental groups; the results are presented as mean ± SD (see Supplementary Material Table S6). * *p* < 0.05; ** *p* < 0.01; *** *p* < 0.001; **** *p* < 0.0001.

Pro-inflammatory cytokines are secreted by Th1 cells, CD4^+^ cells, macrophages, and dendritic cells. Within the plethora of pro-inflammatory cytokines, key functions are sustained by IL-1, IL-6, IFN-g, and TNF-α. These cytokines signal via type I cytokine receptors (CCR1), which are structurally divergent from other cytokine receptor types. They are crucial for coordinating the cell-mediated immune response and play a critical role in modulating the immune system. Pro-inflammatory cytokines generally regulate growth, cell activation, differentiation, and homing of the immune cells to the inflamed sites [55]. In our experimental model, highly significant increased values were obtained in all psoriatic mice, with the highest values obtained in obesity-induced Pso. Interestingly, when obese mice were fed a normal diet afterward, the inflammatory pattern returned to control levels (Figure 5).

**Figure 6:**
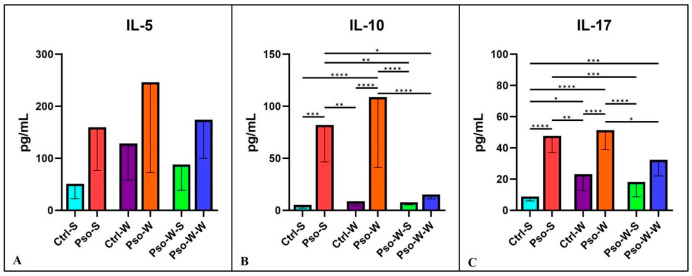
Serum level distribution of IL-5 (**A**), IL-10 (**B**), and IL-17 (**C**) in all experimental groups; the results are presented as mean ± SD (see Supplementary Material Table S6). * *p* < 0.05; ** *p* < 0.01; *** *p* < 0.001; **** *p* < 0.0001.

In our study, the Th-2 cytokines involved in the psoriatic events were IL-5 and 10. Although we did not obtain statistically significant differences between groups in the case of IL-5 (Figure 6A,B), in the case of IL-10, statistically significant differences for both psoriatic groups (Pso-S and Pso-W) were obtained, as compared to their control groups. For IL-17, a cytokine with a crucial role in the pathogenesis of Pso, we also obtained significantly increased serum levels for psoriatic groups as compared to controls (Figure 6C). After standard diet administration to mice with induced obesity and Pso, we observed the normalization of serum levels of these cytokines, and practically no statistically significant differences between the Ctrl-S and Pso-W-S groups were obtained. In the case of IL-10 and IL-17, the psoriatic mechanisms govern the statistically increased circulatory levels rather than the induced obesity.

**Figure 7:**
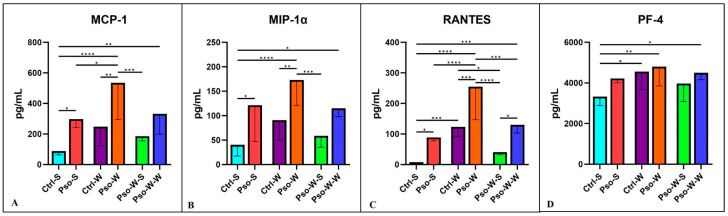
Serum levels distribution of MCP-1 (**A**), MIP-1α (**B**), RANTES (**C**), and PF-4 (**D**) in all experimental groups; the results are presented as mean ± SD (see Supplementary Material Table S6). * *p* < 0.05; ** *p* < 0.01; *** *p* < 0.001; **** *p* < 0.0001.

**Figure 8:**
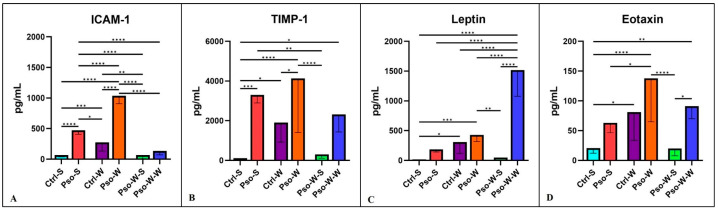
Serum level distribution of ICAM-1 (**A**), TIMP-1 (**B**), Leptin (**C**), and Eotaxin (**D**) in all experimental groups; the results are presented as mean ± SD (see Supplementary Material Table S6). * *p* < 0.05; ** *p* < 0.01; *** *p* < 0.001; **** *p* < 0.0001.

The chemokine landscape in our experimental model shows a statistically significant increment in all the tested chemokines (MCP-1, MIP-1, RANTES, PF-4, and Eotaxin). The highest increase is seen in the psoriatic–obesity group, where we can postulate that chemokine secretion is a cumulative process of both Pso and obesity. Like in the above-described cytokine cases, normalization of the diet induces the lowering of the circulatory levels (Figure 7 and Figure 8).

In our experimental model, we found several other circulatory molecules that were increased mainly in the Pso–obesity group, like ICAM-1 and TIMP-1. Not surprisingly, leptin, an important metabolic hormone [56], was found to be increased in the circulation of the psoriatic–obesity group, especially in the group that was continuously subjected to the W diet (Figure 8).

The profile of the psoriatic–obesity model is profoundly inflammatory in terms of cytokines/chemokines; in most cases, obesity is augmenting the inflammatory pattern of psoriatic events.

### 2.4. Gut Microbiota Assessment in Dietary-Induced Obesity Pso

To determine whether the gut microbiome is altered in conditions of Pso and/or obesity, we employed four diversity metrics to evaluate community richness (Chao1 and observed species) and diversity (Shannon and Simpson) in established mouse models.

**Figure 9:**
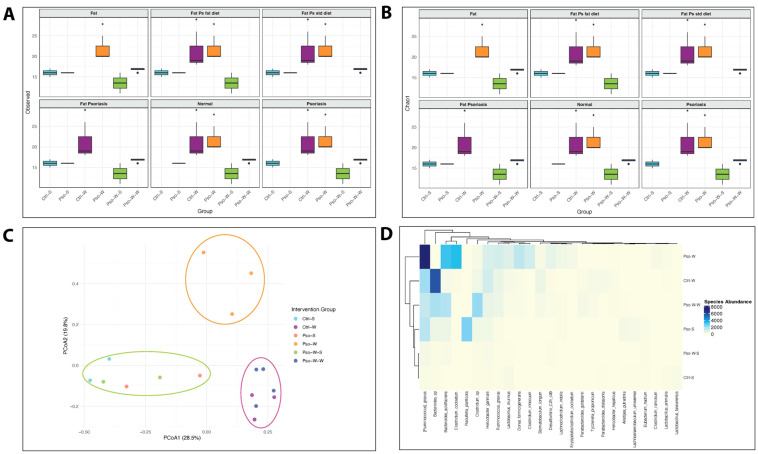
Microbiome community composition and diversity analyses: (**A**) Observed species richness across intervention groups, with leave-one-group-out exclusion strategy shown in facets. Each box represents the alpha diversity distribution within the indicated group after excluding the specified intervention group. (**B**) Chao1 estimator of species richness for the same leave-one-out exclusion design. Colors indicate experimental groups: Ctrl-S (cyan), Pso-S (coral), Ctrl-W (magenta), Pso-W (orange), Pso-W-S (chartreuse), Pso-W-W (blue). (**C**) Principal Coordinate Analysis (PCoA) based on Bray–Curtis dissimilarity of microbial communities, showing clustering by intervention group. Ellipses indicate 95% confidence intervals for group centroids. (**D**) Heatmap of relative abundances, clustered by sample group and taxon. Color scale indicates log-transformed relative abundance. * *p* < 0.05.

Our study found that obesity and combined obesity–psoriatic conditions significantly influence community richness, with higher observed species and Chao1 indices in these conditions compared to normal, psoriatic, and dietary interventional states (all *p* < 0.05) (Figure 9A,B). Interestingly, both observed species and Chao1 index values revealed a biphasic pattern in obesity-enhanced Pso, consisting of an initial increase during the transition from Pso to Pso-W (*p* < 0.05), likely because of rare taxa expression driven by an inflammatory environment. This was followed by a notable decrease in mice on a prolonged high-fat diet (Pso-W vs. Pso-W-W, *p* < 0.05), likely due to the sustained inflammatory and metabolic stress that leads to the selection of a limited number of species that can survive in these environments (Figure 9A,B). In parallel, the administration of a standard diet in Pso-W mouse models results in the reestablishment of bacterial diversity at levels closer to normal. Assessment of diversity metrics such as the Shannon index did not reveal statistically significant differences between experimental groups; however, an opposite trend between Shannon/InvSimpson and Chao1 was observed, highlighting that as microbial richness increases, overall biodiversity decreases in obesity-linked states, which may be a possible hallmark of opportunistic taxa dominance in those settings (Supplementary Materials, Figure S1).

Regarding β-diversity, PCoA ordination based on Bray–Curtis dissimilarities revealed significant differences in microbial composition between psoriasis, obesity, and their co-occurrence at both the species (R^2^ = 0.671, *p* = 0.001) (Figure 9C) and family (R^2^ = 0.634, *p* = 0.001) levels. While psoriasis alone induces relatively modest alterations in bacterial community structure, obesity has a more pronounced effect, with long-term exposure to a high-fat diet resulting in convergence across different pathological states. Yet, the co-occurrence of psoriasis and obesity results in a distinct microbial profile at the species level, likely driven by the overlapping effects of associated inflammatory and metabolic alterations (Figure 9C).

Among the top 25 taxa, differential abundance patterns were evident between the assessed conditions.

Topical administration of IMQ to the mice’s skin induced significant changes in the gut microbiota of Pso mouse models compared to control mice. Therefore, mice in the Pso-S group were characterized by an increase in the *Raoultella*, *Eubacterium*, *Clostridium*, *Citrobacter*, *Lachnoanaerobaculum*, *Lachnoclostridium*, *Alistipes*, *Bacteroides*, and *Faecalibacterium* genera and a pronounced decrease in beneficial *Lactobacillus* strains (*L. murinus* and *L. taiwanensis*) (Supplementary Materials, Figure S2). A possible explanation for the co-occurrence of SCFA producers (e.g., *Faecalibacterium*, *Alistipes*, and *Bacteroides*) with proinflammatory pathobionts such as *Raoultella* or *Clostridium* in the Pso-S group may be the triggering of a resistance mechanism to pathogen colonization resulting from intestinal dysbiosis, as recently described by Yin et al. [57].

In addition, taxonomic analysis identified increased abundances of the genera *Ruminococcus*, *Clostridium*, *Bacteroides*, and *Helicobacter* in the obesity-linked states (Ctrl-W, Pso-W, and Pso-W-W) (Supplementary Materials, Figure S2). The signature of obesity-aggravated Pso (Pso-W) consisted of increased abundances of *Ruminococcus*, *Clostridium*, *Lachnoclostridium*, *Desulfovibrio*, and *Enterorhabdus* genera. However, the transition from Pso-W to Pso-W-W involved a dramatic reduction in the proinflammatory *Ruminococcus* and *Clostridium* genera, concomitant with an increase in beneficial *Alistipes*, *Parabacteroides*, and *Tyzzerella*, leading to the establishment of a microbial community structure comparable to that of the Ctrl-W group.

At the family level, *Lachnospiraceae*, *Erysipelotrichaceae*, and *Bacteroidaceae* were more abundant in the obesity-enhanced Pso group and lower in the normal and Pso-W-S groups (Supplementary Materials, Figure S3). Prolonged high-fat intake resulted in an increase in the *Rikenellaceae*, *Desulfovibrionaceae*, and *Ruminococcaceae* families, whereas dietary normalization alone restored a microbial composition comparable to that of normal control mice. Pso-S was linked to *Enterobacteriaceae* abundance. At the species level, the Ctrl-W group was characterized by elevated levels of *Bacteroides* sp., *Tyzzerella propionicum*, *Parabacteroides distasonis*, and *Helicobacter hepaticus* (Figure 9D). Conversely, the Ctrl-S group exhibited reduced *Bacteroides* sp. levels and was enriched in two *Lactobacillus* strains, namely *L. taiwanensis* and *L. murinus*. Topical application of IMQ on normal mice skin resulted in *Lactobacillus* depletion and increased abundances of *Raoultella planticola*, *Ruminococcus gnavus*, *Bacteroides* sp., *Clostridium* sp., *Helicobacter ganmani*, *Alistipes putredinis*, *Lachnoanaerobaculum umeaense*, and *Eubacterium hadrum* in the gut (Pso-S). Interestingly, *Lactobacillus murinus* levels were restored in the obesity-enhanced Pso group (Pso-W). However, prolonged exposure to a high-fat diet led to a marked reduction in *Lactobacillus murinus*, *Ruminococcus gnavus*, *Bacteroides acidifaciens*, *Clostridium cocleatum*, *Dorea formicigenerans*, *Desulfovibrio_C21_c20*, and *Lachnoclostridium indolis* and a concurrent increase in *Clostridium* sp., *Parabacteroides goldsteinii*, and *Tyzzerella propionicum*, similar to the Ctrl-W group, highlighting obesity as a major driver of gut microbiome alterations in Pso mice.

**Figure 10:**
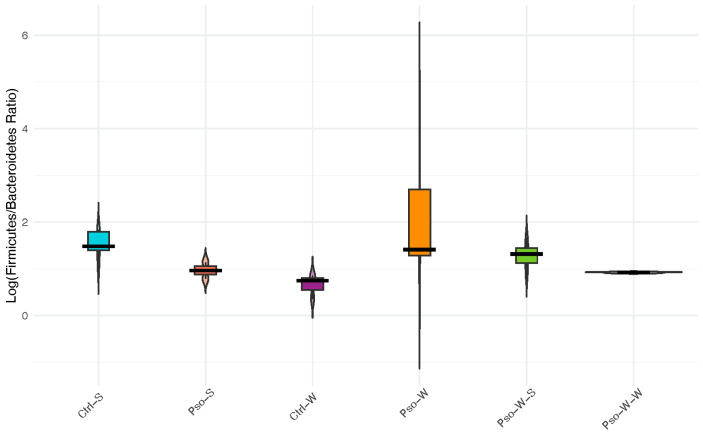
*Firmicutes*/*Bacteroidetes* ratio across intervention groups: Log-transformed Firmicutes-to-*Bacteroidetes* ratios are shown for each experimental group using violin plots overlaid with boxplots. Colors indicate intervention groups: cyan (Ctrl-S), coral (Pso-S), dark magenta (Ctrl-W), orange (Pso-W), chartreuse (Pso-W-S), and blue (Pso-W-W). Wider violins indicate greater density of values. Differences in central tendency and distribution reflect group-specific shifts in relative abundance of these dominant phyla.

The ratio between *Firmicutes* and *Bacteroidetes* phyla (F/B ratio), which represents more than 90% of the total gut microbiota community, has recently emerged as a potential indicator of normal intestinal homeostasis, as changes in this ratio may reflect the onset of certain pathologies, including obesity. Here, we report no statistically significant changes in the F/B ratio across the investigated groups, although a decrease in *Firmicutes* and an increase in *Bacteroidetes* abundance were observed in the Pso-S and Ctrl-W groups compared to normal mice (Figure 10). Combined Pso and obesity conditions (Pso-W) induced a shift in gut microbiota compared to the associated control groups (Pso-S, Ctrl-W), which resulted in a slight increase in *Firmicutes* and the F/B ratio, but this trend was not maintained under high-fat dietary conditions.

### 2.5. Evaluating the Relationship Between Gut Microbiota and Host Inflammation

To identify whether the identified taxa are correlated with obesity-linked inflammation, a correlation analysis of microbiome and protein array data was performed using Spearman’s correlation coefficient.

**Figure 11:**
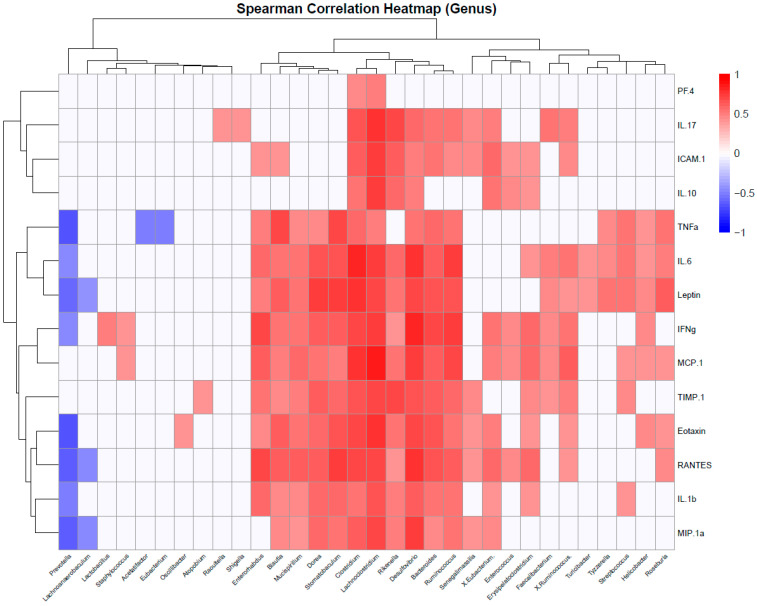
Heatmap of Spearman correlations between bacterial genera and host cytokine levels. The heatmap shows Spearman correlation coefficients between the relative abundances of bacterial genera (columns) and circulating cytokine levels (rows). Positive correlations are indicated in red and negative correlations in blue, with color intensity reflecting correlation strength (scale from −1 to +1). Hierarchical clustering of rows and columns highlights patterns of shared associations among bacterial genera and immune markers.

At the genus level, pro-inflammatory cytokines (e.g., TNFα, IL-6) were positively associated with *Helicobacter*, *Clostridium*, Blautia, *Mucispirillum*, *Dorea*, *Stomatobaculum*, *Lachnoclostridium*, *Rikenella*, *Desulfovibrio*, *Bacteroides*, *Tyzzerella*, *Streptococcus*, *Roseburia*, and *Ruminococcus* (Figure 11). Interestingly, leptin, the only analyte that remained elevated during prolonged exposure to a high-fat diet, maintained the same correlation patterns, indicating similarities between the Ps-W and Ps-W-W groups in terms of microbiome–inflammation dynamics. IL-17, a cytokine particularly linked to Pso in our study, was correlated with increased Raoultella and Shigella abundances, among others. In contrast, *Prevotella*, *Lachnoanaerobaculum*, *Acetatifactor*, and *Eubacterium* negatively correlated with indicators of obesity-associated inflammation, suggesting that members of these genera may contribute to the attenuation of metabolic dysfunction in Pso mouse models.

Next, we were interested in which families and species were associated with the inflammation biomarkers.

At the family level, *Lachnospiraceae*, *Erysipelotrichaceae*, and *Bacteroidaceae*, which were enriched in the obesity-related groups, either with or without Pso, displayed positive correlations with all the investigated proinflammatory cytokines and chemokines, except IL-10 and PF4 (Supplementary Materials, Figure S4). Interestingly, leptin and IL-6 were the only factors associated with *Cohaesibacteraceae* and *Rhodospirillaceae* families, highlighting additional microbial signatures for obesity-enhanced psoriasis. In addition, increased abundances of *Enterobacteriaceae*, associated with the Pso-S groups, were found to be positively correlated with ICAM1, TIMP-1, IL-1β, and IFNγ.

At the species level, we observed that proinflammatory factors TNFα, IL-6, IL-1β, IFN-γ, IL-17, and leptin established positive correlations with *Desulfovibrio_C21_c20*, *Clostridium* sp., *Ruminococcus gnavus*, *Dorea formicigenerans*, *Parabacteroides goldsteinii*, *Tyzzerella propionicum*, *Helicobacter hepaticus*, *Lactobacillus murinus*, *Bacteroides acidifaciens*, and *Stomatobaculum longum*, among others. Yet, negative correlations between these proinflammatory factors were found for the taxa *Lactobacillus taiwanensis*, *Alistipes putredinis*, *Eubacterium hadrum*, *Lactobacillus johnsonii*, and *Ruminococcus gauvreauii*, which may represent potential taxa for intervening in obesity-aggravated psoriasis.

### 2.6. Evaluating the Relationship Between Gut Microbiota and Host Metabolism Parameters

Since dietary context modulates microbial composition, resulting in altered immune and metabolic states, we evaluated the microbial taxa that were significantly correlated with cholesterol and triglyceride levels, as well as body and spleen weight, to identify potential biomarkers for disease severity and therapeutic interventions in our mouse models.

At the genus level, *Bacteroides*, *Ruminococcus*, *Helicobacter*, and *Clostridium*, which were more abundant in the obesity-related groups, showed, as expected, positive correlations with cholesterol levels (Supplementary Materials, Figure S5). Additional genera, such as *Parabacteroides*, *Tyzzerella*, *Roseburia*, *Enterorhabdus*, *Dorea*, *Stomatobaculum*, and *Blautia*, were also linked to increased levels of this metabolic parameter. In addition, splenomegaly, a particular hallmark of Pso, exhibited positive correlations with *Lachnoclostridium*, *Clostridium*, *Desulfovibrio*, *Raoultella*, and *Citrobacter*. Conversely, higher abundances of *Eubacterium*, *Prevotella*, and *Lachnoanaerobaculum* were linked to lower body weight, TG, and cholesterol levels, as well as reduced splenomegaly, suggesting potential microbial signatures linked to lipid metabolism and disease severity in our Pso mice models.

At the family level, most inflammation-associated taxa (e.g., *Erysipelotrichaceae*, *Xanthomonadaceae*, *Bacteroidaceae*, *Desulfovibrionaceae*, *Lachnospiraceae*, *Ruminococcaceae*, *Clostridiaceae*, and *Rikenellaceae*) displayed positive correlations with body weight and cholesterol levels, underscoring their potential as biomarkers of disease severity (Supplementary Materials, Figure S6). Concomitantly, higher abundances of all the investigated families were related to lower TG levels. Yet, increased *Flavobacteriaceae* and *Flammeovirgaceae* levels tend to improve metabolic health, leading to a reduction in cholesterol levels. In addition, spleonomegaly was positively impacted by increased levels of *Erysipelotrichaceae*, *Xanthomonadaceae*, *Lachnospiraceae*, *Cytophagaceae*, *Eubacteriaceae*, and *Enterobacteriaceae* and negatively by *Flavobacteriaceae* and *Neisseriaceae* families.

**Figure 12:**
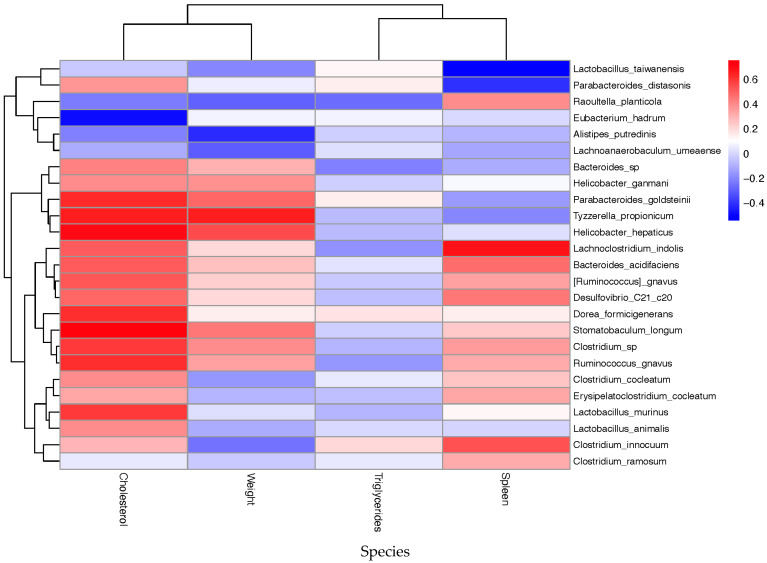
Heatmap of Spearman correlations between bacterial species (species level) and host metabolic parameters. The heatmap displays Spearman correlation coefficients between log-transformed relative abundances of bacterial species (rows, species-level resolution) and host clinical variables (columns): cholesterol, weight, triglycerides, and spleen weight. Positive correlations are shown in red and negative correlations in blue, with color intensity indicating the strength of association. Hierarchical clustering of rows and columns highlights groups of taxa and host traits with similar correlation patterns.

Extended species-level correlation analysis revealed positive associations between elevated cholesterol levels and most taxa implicated in inflammation, such as *Tyzzerella propionicum*, *Helicobacter hepaticus*, *Dorea formicigenerans*, *Stomatobaculum longum*, *Clostridium sp*, and *Ruminococcus gnavus* (Figure 12). In contrast, elevated levels of *Lactobacillus taiwanensis*, *Eubacterium hadrum*, and *Alistipes putredinis* have shown potentially beneficial effects in attenuating the obesity burden through the reduction in cholesterol concentration and body weight. Aggravated psoriasis symptomatology, derived from splenomegaly, demonstrated positive correlations with *Lachnoclostridium indolis*, *Bacteroides acidifaciens*, *Ruminococcus gnavus*, *Raoultella planticola*, *Desulfovibrio_C21_c20*, and *Clostridium innocuum* and negative associations with *Lactobacillus taiwanensis* and *Parabacteroides distasonis*. Therefore, all these data suggest that increased abundances of *Lactobacillus taiwanensis*, *Alistipes putredinis*, and *Eubacterium hadrum* may be beneficial to alleviate the inflammatory and metabolic burden in obesity-aggravated Pso mice models.

## 3. Discussion

Given that dietary context can significantly influence gut microbial composition—thereby modulating immune responses and metabolic outcomes—we explored the interplay between microbial signatures and inflammatory mediators. Our goal was to uncover novel microbiome-targeted strategies for addressing obesity-aggravated Pso in an IMQ-induced mouse model.

In the last two years, there have only been seven published papers regarding the study of microbiota in IMQ-induced Pso, with even fewer on obesity-induced experimental psoriasiform dermatitis, but there have been several studies reported on Pso patients. Using a large-scale GWAS (genome-wide association study) and two-sample Mendelian randomization, a recent study evaluated the association between gut microbiota make-up and Pso. The authors showed a protective role of *Bacteroidetes* and *Prevotella* in Pso, while the *E. fissicatena* group was a possible risk factor for Pso [56]. Another study identified *Lactococcus*, *Ruminiclostridium*, and *Eubacterium fissicatena* in Pso patients as risk factors, while *Odoribacter* demonstrated a protective effect [58], and another study linked Pso risk to *Mollicutes*, *Victivallaceae*, *Eubacterium* (*Coprostanoligenes* and *Fissicatena* groups), *Holdemania*, *Lachnospiraceae*, *Lactococcus*, and *Tenericutes* [59]. As shown, there is a diversity in the microbiota pattern associated with Pso. In our experimental model, increased abundances of *Raoultella* and *Eubacterium* were the main risk factors identified for Pso development. The pathological relevance of the genus *Raoultella*, along with other members of the *Enterobacteriaceae* family, is being recognized due to its negative association with short-chain fatty acid (SCFA) producers [57]. It seems that in pathological settings, dysbiosis may lead to a decrease in commensals and SCFA producers, compromising the antibacterial immune mechanisms and favoring the expansion of pathogens such as *Enterobacteriaceae*, which are able to produce metabolites associated with oxidative stress and inflammation [60,61]. In addition, although the majority of *Eubacterium* species are butyrate producers [62], current evidence on how their increase might influence Pso risk is conflicting; this is further complicated by the fact that some species, such as *E. hadrum*, have been recently reassigned to other genera (e.g., *Anaerostipes*) based on their distinct and hence not fully understood metabolic features [56,63].

In addition, Pso’s association with a continuous Western diet induced a predominance of *Ruminococcus*, *Clostridium*, and *Desulfovibrio*—genera often linked to inflammation—while the Ctrl-W and Pso-W-S groups showed higher levels of *Bacteroides*, *Prevotella*, and *Blautia*, taxa associated with microbial stability and SCFA production. Interestingly, upon reversing the diet to a normal one, the levels of inflammation-prone bacteria drop in favor of non-inflammatory ones. The corresponding study of Wen et al. on the mouse model showed that the relative levels of gut microbiota are different compared to healthy controls. *Firmicutes* and *Bacteroidetes*’ relative abundances were reported as reversed, along with *Escherichia coli*, which was found to be significantly enriched in the Pso group [64]. Our experimental model confirms the involvement of *Enterobacteriaceae* and *Eubacteriaceae* members in Pso pathophysiology, as well as reversed *Firmicutes* and *Bacteroidetes*’ abundances, mainly controlled by the fatty diet and influenced positively by normalization of the diet.

As mentioned in the introduction, one of the modalities of dysbiosis installation is microbiota-derived molecules (metabolites). These metabolites have seminal importance in immune cells and the cytokine/chemokine disbalance in Pso [65]. In the present study, we conducted a correlation analysis to identify key microbial taxa associated with host inflammation and lipid metabolism. The proinflammatory factors TNFα and IL-6 were positively associated with *Clostridium* sp., *Ruminococcus gnavus*, and *Tyzzerella propionicum*, among others. In contrast, taxa such as *Lactobacillus taiwanensis*, *Alistipes putredinis*, *Eubacterium hadrum*, *Lactobacillus johnsonii*, and *Ruminococcus gauvreauii* were negatively correlated with inflammatory markers, suggesting potential anti-inflammatory effects. Interestingly, the same pathobionts involved in inflammation displayed positive correlations with metabolic stress indicators (e.g., body weight, cholesterol, triglycerides, and splenomegaly), whereas commensals such as *L. taiwanensis*, *A. putredinis*, and *E. hadrum*, known for producing beneficial metabolites, showed inverse associations. Therefore, taken together, all these data suggest that *L. taiwanensis*, *A. putredinis*, *E. hadrum*, *L. johnsonii*, and *R. gauvreauii* might be potential taxa for attenuating the inflammation and metabolic burden in obesity-enhanced Pso. At the same time, we consider that the overrepresented taxa linked to inflammation and obesity may be envisioned as possible targets for metabolic interventions in obesity-aggravated psoriasis.

Methods to therapeutically intervene in gut microbiota regulations have also been published. Thus, in a 12-week, open-label, single-center clinical trial, patients receiving anti-psoriatic local therapy in combination with probiotic and prebiotic supplementation had an overall objective improvement in their clinical parameters [66]. In a similar study involving Southern Chinese psoriatic patients with elevated levels of *Blautia wexlerae* and *Parabacteroides distasonis*, an 8-week administration of a complete formula of prebiotics, probiotics, and postbiotics led to improvements in clinical symptoms and normalization of the gut microbiome [67]. In one of our previous studies, Surcel et al. showed in a similar animal model of Pso that orally administered IgY targeting gut-deregulated microbiota populations can significantly alleviate the Pso pattern and inflammatory immune actors [68]. However, in the current Pso mouse model, we intervened in the bacterial consortium only by normalization of the diet and, much to our surprise, the mere lowering of the fat component normalized the gut landscape.

Thus, our study emphasizes certain microbial taxa that may be used to alleviate the inflammation burden in obesity-aggravated Pso. Among them are some *Lactobacillus* strains, which may be employed as probiotics/postbiotics to modulate gut homeostasis. Lamas et al. highlighted the potential of three *Lactobacillus* strains, including *L. taiwanensis*, to attenuate intestinal inflammation in a mouse model of colitis. The beneficial effects exerted by *Lactobacillus* strains consisted of their ability to metabolize tryptophan into compounds that act as aryl hydrocarbon receptor (AhR) ligands, leading to AhR activation and rescue of IL-22 production, which is critical for microbiome stability [69]. In parallel, other species such as *L. johnsonii* have been shown to improve gut microbiota composition by exerting anti-biofilm properties (impeding the pathobionts from expanding) and supporting the proliferation of SCFA-producing bacteria [70]. Another study recently highlighted the ability of *L. johnsonii* to produce high levels of SCFAs, which have been effective against *Salmonella* infections and other pathogens in the intestines of pigs [71]. Moreover, post-natal administration of *L. johnsonii* in T1D-prone rats was reported to regulate the structure of the intestinal barrier, reducing intestinal inflammation by increasing the expression of claudin-1 and decreasing the expression of occludin [72]. In addition, in a mouse model of colitis, *L. johnsonii* has been shown to alleviate the associated symptomatology through the activation of anti-inflammatory macrophages and stimulation of IL-10 production via the TLR1/2-STAT3 pathway [73].

*Alistipes putredinis*, a member of the *Rikenellaceae* family, is another commensal with documented beneficial effects on the intestinal barrier and immune function. Several studies reported the involvement of *A. putredinis* in regulating body weight and inflammation, both in human subjects and animal models [74,75]. Yet, Zhang et al. have recently proven that, besides attenuating inflammation and gut dysbiosis, *A. putredinis* regulates lipid accumulation in rat models of high-fat diet-induced metabolic dysfunction-associated steatotic liver disease, strengthening the assertion that it may be a beneficial taxon in the context of obesity-enhanced Pso [76]. *Ruminococcus gauvreauii*, an SCFA producer from the *Lachnospiraceae* family, might be another promising taxon to attenuate the metabolic burden in obesity-enhanced Pso, taking into account the fact that SCFAs can modulate the balance between Tregs and Th17 cells, which is critical in autoimmunity [77]. However, conflicting results suggesting concomitant pro-inflammatory roles of these bacteria urge caution regarding their pharmacological use in clinical settings [78]. The same observations can be applied to the butyrate-producing *Eubacterium hadrum*, which has been reassigned to the *Anaerostipes* genus (*A. hadrus*), as it seems to be a risk factor for Pso in our experimental mouse model [62,63].

Our study has several limitations, primarily related to the IMQ-induced mouse model chosen, which does not reflect the chronic and intricate pathophysiology of human Pso [79], and to the low number of metagenomic samples. Moreover, our study provides associations between microbial taxa and indicators of inflammation and obesity, but does not establish causation. Causation analysis implies longitudinal studies [80], which were beyond the scope of this experiment, but may represent an important research direction in the future. Despite these limitations, our study makes a valuable contribution to the field by providing potential microbial signatures and accessible interventional strategies for obesity-aggravated Pso in animal models, partially sustained by other studies. Therefore, these findings highlight the need to expand the research on human cohorts, as Pso and obesity are two closely interrelated conditions associated with significant socio-economic and psychological burdens worldwide.

In summary, gut microbiota network analysis in both experimental models and human cases of Pso reveals a disrupted yet interconnected microbial ecosystem, suggesting cross-border homeostasis among microbial populations under inflammatory conditions. Despite growing insights, the specific genera and species involved in Pso, along with their intricate interactions with the host immune system, remain only partially understood. Further investigation is essential to clarify these relationships, which may open new avenues for microbiome-based therapeutic strategies targeting inflammation and metabolic dysfunction in psoriasis [81].

## 4. Materials and Methods

### 4.1. Experimental Model

Animals. C57BL/6 mice (Jackson Laboratory, Bar Harbor, ME, USA), 14-week-old males and females, were provided by the Animal Husbandry from Victor Babeș National Institute. The animals (fed and watered ad libitum) were housed individually in special cages and maintained in optimal conditions of temperature (20 ± 4 °C), humidity (5 ± 10%), artificial ventilation, and lighting (with a 12/12 light/dark cycle). All cages were maintained under a rigorous cleaning and sanitation program, and the health status of the animals was monitored daily. Metabolic cages were used to collect fecal samples.

**Figure 13:**
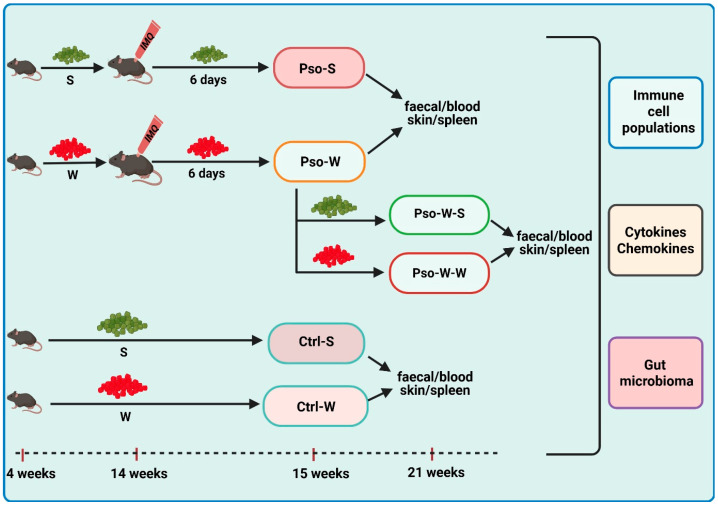
The outline of the experimental model. The graphical scheme includes the main key points of the study and the involved experimental groups (Created in https://BioRender.com).

IMQ-induced Pso model and diet-induced obesity. The IMQ-based murine model was replicated according to the protocols described in the literature [82]. For the group subjected to dietary-induced obesity, mice were fed a Western diet (W) (Fat diet C1090-45 obesity-inducing diet with w/45% energy from fat, Altromin International, Lage, Germany), while control diet groups were fed standard Purina 5L79 rodent chow (S) (Agripurina, Warburg, AB, Canada). The six experimental groups were as follows: (1) Pso-S: 8 mice (aged 14 weeks, sex ratio 1:1) received a daily topical dose of 62.5 mg IMQ-based cream (5% Aldara Cream, MEDA AB, Solna, Sweden) on the shaved back for 6 consecutive days and were sacrificed on day 7; feeding was done with S; (2) Pso-W: 18 mice (aged 4 weeks, sex ratio 1:1), prior to establishing the IMQ model, were fed for 10 weeks with W; after 10 weeks, they received a daily topical dose of IMQ, as previously described, in order to develop Pso. Of these, 6 mice were sacrificed on day 7, and the rest of them were reverted to S (Pso-W-S group—6 mice) (3) or continued to W (Pso-W-W group—6 mice) (4) for another 6 weeks, and both groups were sacrificed in week 21. The controls were (5) Control (Ctrl)-W group—8 mice (age 4 weeks, sex ratio 1:1) fed with W for 10 weeks and sacrificed in week 15, and (6) Ctrl-S group—8 mice (age 15–16 weeks, sex ratio 1:1) fed with S. For all experimental groups, the body weights of the animals were recorded weekly (Scientech SL 3100D, Boulder, CO, USA). A graphical outline of the experimental model is depicted in Figure 13.

Skin inflammation assessment was performed for all IMQ groups, whether fed S or W diets. Daily PASI scores (0–12 scale) were obtained by summing the scores of erythema, thickening, and scaling on a 0–4 scale (0—no change, 1—mild change, 2—marked change, 3—significant change, 4—severe change).

Biological sample collection and sample processing. In weeks 15 and 21, the animals were anesthetized with the ketamine/acepromazine/xylazine cocktail (ketamine 80 mg/kg, Richterpharma AG, Wels, Austria; acepromazine 6 mg/kg, Vetoquinol SA, Lure, France; xylazine 1 mg/kg, Bioveta SA, Ivanovice na Hané, Czech Republic), and blood, spleen, and skin samples were collected. Peripheral blood was collected by an intracardiac puncture in K2-EDTA-coated tubes (SARSTEDT AG & CO. KG, Nümbrecht, Germany) for immune cell evaluation (flow cytometry) and cytokine/chemokine/growth factor evaluation (protein microarray). Cholesterol and Triglycerides were evaluated from peripheral blood using specific Accutrend test strips (Accutrend Plus Instrument Roche Diagnostics, Mannheim, Germany). For splenomegaly evaluation, spleens were weighed (Balance AEP-1500A, Adam Equipment Co., Ltd., Kingston, Milton Keynes, UK). Skin samples were processed (fixed in 10% buffered formalin, embedded in paraffin, and sectioned in 5 μm sections for hematoxylin and eosin [H&E] staining) prior to histopathological evaluation (Olympus BX43 with CellSens Dimension Program, Tokyo, Japan). Fecal samples were antiseptically collected at weeks 15 and 21 and were immediately frozen at −80 °C until microbiota evaluation.

### 4.2. 16S rRNA Sequencing

**Figure 14:**
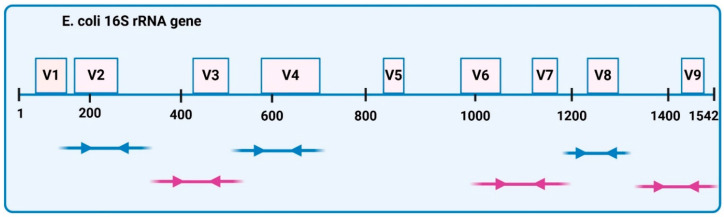
Schematic *E. coli* 16S rRNA gene and primer targets. The 16S rRNA gene is about 1542 base pairs long and includes nine hypervariable regions (V1–V9) along with conserved regions. Ion 16S Metagenomics kit contains two sets of primers targeting seven hypervariable regions (V2-4-8 and V3, V6-7, V9) (blue arrows—primers for V2-4-8 regions, purple arrows—primers for V3, V6-7, V9 regions). Created in https://BioRender.com, adapted from ThermoFisher Scientific, Inc. [83].

Twenty-four fecal samples were included in the 16S rRNA sequencing analysis, as follows: Ctrl-S (*n* = 6), Ctrl-W (*n* = 3), Pso-S (*n* = 2), Pso-W (*n* = 3), Pso-W-S (*n* = 5), and Pso-W-W (*n* = 5). Microbial genomic DNA was extracted using the PureLink Microbiome Purification Kit (Invitrogen, Waltham, MA, USA), which enables fast extraction of high-quality microbial DNA. The concentration and purity of the DNA were quantified using a NanoDrop 2000 spectrophotometer (ThermoFisher Scientific, Waltham, MA, USA), the Qubit dsDNA HS Assay Kit, and the Qubit 2.0 fluorimeter (ThermoFisher Scientific, Waltham, MA, USA). All the samples of bacterial genomic DNA were diluted to 2 ng/mL, and for library preparation, 2 µL per probe was used. Microbial DNA libraries were prepared using the Ion 16S Metagenomics Kit (ThermoFisher Scientific, Waltham, MA, USA). Amplification was performed using two primer pools to amplify seven hypervariable regions (V2-4-8 and V3, V6-7, V9) of bacterial *E. coli* 16S rRNA (Figure 14). High-quality DNA Libraries were generated using the Ion Plus Fragment Library kit (ThermoFisher Scientific, Waltham, MA, USA). After amplification, PCR products were purified with Agencourt AMPure beads (Beckman Coulter, Brea, CA, USA) to remove primer dimers and end-repaired for barcode ligation with the Ion Xpress Barcode Adapters 1-16 Kit. Library concentrations were quantified using the Ion Universal Library Quantitation kit (ThermoFisher Scientific, Waltham, MA, USA) and adjusted to a 10pM concentration each. Equal volumes of all samples were combined and processed for template preparation using the ION PGM Hi-Q OT2 kit (ThermoFisher Scientific, Waltham, MA, USA) and Ion OneTouch 2 System (ThermoFisher Scientific, Waltham, MA, USA). Sequencing of the amplicon libraries was carried out on 316 Chip Kit v2 according to the supplier’s instructions, using the Ion Torrent Personal Genome Machine (PGM) (ThermoFisher Scientific, Waltham, MA, USA). All sequencing reagents were provided by ThermoFisher Scientific (Waltham, MA, USA), except Agencourt AMPure beads.

Sequencing data analysis. Basic analysis was performed using Torrent Suite Software version 5.18 (ThermoFisher Scientific, Waltham, MA, USA) with default parameters. The individual sequence reads were filtered with Ion Reporter Software version 5.20.8.0 to remove polyclonal sequences and low-quality reads (sequencing data analysis is described in the Section “Microbiome Evaluation Statistics”).

### 4.3. Flow Cytometry

Peripheral blood lymphocyte immunophenotyping included total T lymphocytes (CD3ε^+^), T helper (CD4^+^CD8^−^), T suppressor/cytotoxic (CD8a^+^CD4^−^) subsets, B lymphocytes (CD3ε^−^CD19^+^), and NK cells (CD3ε^−^NK1.1^+^) and was performed via flow cytometry (BD FACSCanto II cytometer, BD Biosciences, San Jose, CA, USA). The staining protocol for surface markers was performed as briefly described: blood samples were first incubated on ice with TruStain fcX (anti-mouse CD16/32, isotype Rat IgG2a, λ) Antibody (BioLegend, San Diego, CA, USA) in order to block non-specific antibody binding, then stained with the following monoclonal antibodies conjugated with fluorochromes: 0.5 μL Alexa Fluor 647 anti-mouse CD3ε (clone 145-2C11, isotype Armenian Hamster IgG, BioLegend, San Diego, CA, USA); 0.5 μL Alexa Fluor 488 anti-mouse CD8a (clone 53–6.7, isotype Rat IgG2a, κ); 1.25 μL PE-Cy7 anti-mouse CD4 (clone GK1.5, isotype Rat IgG2b, κ); 1.25 μL PerCP-Cy5.5 anti-mouse CD19 (clone 6D5, isotype Rat IgG2a, κ); 1.25 μL PE anti-mouse NK1.1 (clone PK136, isotype Mouse IgG2a, κ) (all from BioLegend, San Diego, CA, USA). After red blood cells lysis (BD FACS Lysing Solution, BD Biosciences, San Jose, CA, USA) and two washing steps (Cell Staining Buffer, BioLegend, San Diego, CA, USA), the samples were acquired using a BD FACSCanto II cytometer (BD Biosciences, San Jose, CA, USA) and the data were analyzed with BD FACSDiva v 6.1 software. Data acquisition was preceded by a daily check-up of cytometer performances (BD Cytometer Setup and Tracking Beads Kit, BD Biosciences, San Jose, CA, USA) and compensation of spectral overlaps (UltraComp eBeads, Invitrogen by Thermo Fischer Scientific, San Diego, CA, USA). Unlabeled cells were used as a negative control.

### 4.4. Protein Microarray

Quantification of circulating inflammatory cytokines/chemokines/growth factors/hormones was performed with Quantibody^®^ Mouse Inflammation Array 1 (RayBiotech, Peachtree Corners, GA, USA) and analyzed with MAPIX software 8.2.5 version on an InnoScan 1100AL scanner (Innopsys, Carbone, France). The kit allows the simultaneous analysis of 40 plasma mouse analytes: IL-1α, IL-1β, IL-2, IL-4, IL-5, IL-6, IL-7, IL-10, IL-12p70, IL-15, IL-17A, IL-21, B-Lymphocyte Chemoattractant (BLC/CXCL13), CD30 Ligand (TNFSF8), Eotaxin-1 (CCL11), Eotaxin-2 (MPIF-2/CCL24), Fas Ligand (TNFSF6), Granulocyte-Colony Stimulating Factor (G-CSF), Granulocyte-Macrophage Colony-Stimulating Factor (GM-CSF), I-309 (TCA-3/CCL1), Intercellular Adhesion Molecule-1 (ICAM-1/CD54), IFN-γ, Keratinocyte-Derived Chemokine (KC/CXCL1), Leptin, Lipopolysaccharide-Induced CXC Chemokine (LIX), Monocyte Chemoattractant Protein-1 (MCP-1/CCL2), MCP-5, Macrophage Colony-Stimulating Factor (M-CSF), Monokine Induced by IFN-γ (MIG/CXCL9), Macrophage Inflammatory Proteins (MIP-1α/CCL3), MIP-1 gamma, Platelet Factor 4 (CXCL4), Regulated upon Activation, Normal T cell Expressed and presumable Secreted (RANTES/CCL5), Thymus and Activation Regulated Chemokine (TARC/CCL17), Tissue Inhibitor of MetalloProteinases (TIMP-1), TNF-α, TNF RI (TNFRSF1A), and TNF RII (TNFRSF1B).

### 4.5. Statistics

Data are presented as mean values accompanied by their respective standard deviations (SD). Statistical analyses were performed using Microsoft Excel (Microsoft Corporation, Redmond, WA, USA) and GraphPad Prism version 10.5.0 (GraphPad Software, San Diego, CA, USA). Comparisons between two experimental groups were conducted using the two-tailed Student’s *t*-test, assuming equal variance, with statistical significance defined as *p* < 0.05. For analyses involving more than two groups, data were assessed using ordinary one-way analysis of variance (ANOVA), followed by Tukey’s multiple comparison test to adjust for post hoc pairwise testing (* *p* ≤ 0.05, ** *p* ≤ 0.01, *** *p* ≤ 0.001, **** *p* ≤ 0.0001).

#### Microbiome Evaluation Statistics

Microbial α-diversity across intervention groups was assessed using four widely adopted ecological metrics: Observed Richness, Shannon Diversity Index, Inverse Simpson Index, and the Chao1 Estimator. These indices were computed using the estimate richness function in the *phyloseq* R package, R version 4.1.2. Samples with insufficient sequencing depth (<1000 reads) or incomplete metadata were excluded to ensure data quality. Group-level differences in α-diversity were evaluated with the non-parametric Wilcoxon rank-sum test, chosen for its robustness to non-normal distributions and heteroscedasticity, which are common in ecological datasets. Pairwise comparisons were performed using the *compare_means* function from the *ggpubr* package, version 0.6.1. To examine the stability of these group differences, a sensitivity analysis was conducted by iteratively excluding one intervention group at a time. In each iteration, diversity metrics were recalculated, and the Wilcoxon test was reapplied to identify any group exerting disproportionate influence on overall patterns. The results were visualized with *faceted boxplots*, with significant differences annotated using asterisks (*p* < 0.05). Taxonomic composition at the family, genus, and species levels was visualized using heatmaps of the 25 most abundant taxa. Taxonomic profiles were derived from processed sequencing data, with sample labels standardized using consistent, abbreviated intervention codes. Group-level relative abundances were averaged to produce an abundance matrix for heatmap construction, generated using the *ComplexHeatmap* R package (version 2.241). β-diversity, representing community-level differences in composition, was evaluated using Bray–Curtis dissimilarity, which accounts for both presence/absence and relative abundance data. Control samples and those with a sequencing depth below 1000 reads were excluded prior to analysis. *Phyloseq* objects were constructed at the family, genus, and species levels. Ordination was performed using Principal Coordinate Analysis (PCoA) via the ordinate function in *phyloseq* (version v1.38.0), with axes labeled to indicate the percentage of explained variance. Differences in community composition among groups were tested using Permutational Multivariate Analysis of Variance (PERMANOVA), implemented with 999 permutations via the *adonis2* function in the vegan package (version 2.5-7), with R^2^ values and *p*-values reported.

The *Firmicutes*-to-*Bacteroidetes* (F/B) ratio, a widely used indicator of microbial community imbalance, was calculated using genus-level relative abundance data. Phylum-level assignments were based on established taxonomic references. Ratios were computed per sample and log-transformed using log1p to reduce skewness and stabilize variance. Associations between gut microbial composition and host physiological parameters were explored using correlation analyses at the genus, family, and species levels. The 25 most abundant taxa at each taxonomic resolution were selected based on cumulative relative abundance to reduce noise from rare taxa. Spearman’s rank correlation was used to evaluate monotonic relationships between microbial abundances and host phenotypic traits, including body weight, serum cholesterol, triglyceride levels, and spleen weight. This non-parametric method was chosen for its robustness to non-normality. Correlations were calculated using complete-case analysis, such that samples with missing data were excluded only from analyses involving the missing variables, preserving statistical power while minimizing data loss. Microbial and cytokine datasets were aligned using shared sample identifiers. Cytokine concentrations (*n* = 14) were standardized, and only samples with complete overlap between microbiome and cytokine measurements were retained. Spearman correlations were computed between cytokine levels and microbial abundances across all taxonomic levels. Associations were considered statistically significant at *p* < 0.05 with an absolute Spearman’s ρ (|ρ|) > 0.3. Significant correlations were visualized using heatmaps generated with the *pheatmap* R package, version 1.0.13, employing a diverging color palette to illustrate the strength and direction of associations.

## 5. Conclusions

In recent years, an increasing number of studies have highlighted the important role of the intestinal microbiome in modulating and maintaining various pathologies, including skin disorders, such as psoriasis. The gut–skin axis is regulated by the immune system, with the intestinal microbiome playing an important role in the functionality of the immune system, regulating the homeostasis of this system and intervening at all levels of the components of the innate and adaptive immune system. In our experimental model, we have shown that a Western high-fat diet can induce dysbiosis of the microbiota and that the mere normalization of the diet can alleviate psoriatic development. Targeting the microbiota through diet may represent a new stage in personalized therapy and hope for many psoriatic patients.

## Figures and Tables

**Table 1:** Skin inflammation parameter comparison between Pso mice fed the S (*n* = 8) and W diets (*n* = 18); *n* = number of mice; NS = not statistically significant.

Parameter	*p*-Values for the Pso-S vs. Pso-W Groups			
	Day 1–4	Day 5	Day 6	Day 7
Erythema	NS	NS	2.3 × 10^−7^	5.8 × 10^−5^
Skin scaling	NS	2.4 × 10^−4^	1.6 × 10^−5^	8.5 × 10^−7^
Thickness	NS	2.9 × 10^−2^	1.7 × 10^−2^	1.3 × 10^−6^
PASI score	NS	1.1 × 10^−3^	5.9 × 10^−13^	9.4 × 10^−9^

## Data Availability

The raw 16S rRNA sequencing data generated during this study are available under NCBI BioProject Accession Number PRJNA1284851. Details related to bioinformatics analysis of gut microbiome, cytokine, and metabolic data are available from the corresponding author (P.I.) upon reasonable request.

## Supplementary Materials

The following supporting information can be downloaded at: https://www.mdpi.com/article/10.3390/ijms26167697/s1.
