# Supplementary material for: Microbial Signatures of Obesity-Aggravated Psoriasis: Insights from an Imiquimod-Based Mouse Model
Source: Int J Mol Sci. 2025 Aug 8;26(16):7697. doi: 10.3390/ijms26167697 (PMC12386422; doi:10.3390/ijms26167697)
Supplement: Supplementary file 1 [file ijms-26-07697-s001.zip › ijms-3800715-supplementary.pdf]

Supplementary material

Table S1. Mean values of inflammatory parameters in Pso-S and Pso-W groups (mean±SD)

		Day 1	Day 2	Day 3	Day 4	Day 5	Day 6	Day 7
**Erythema**	**Pso-S**	0.0±0.0	1.10±0.2	1.90±0.2	2.55±0.4	3.15±0.2	3.05±0.3	2.70±0.3
	**Pso-W**	0.0±0.0	1.2±0.3	2.03±0.3	2.69±0.3	3.31±0.3	3.78±0.3	3.31±0.3
**Skin**	**Pso-S**	0.0±0.0	0.0±0.0	1.05±0.2	1.50±0.2	1.95±0.2	2.60±0.3	3.05±0.3
**scaling**	**Pso-W**	0.0±0.0	0.32±0.3	1.06±0.2	1.56±0.3	2.31±0.2	3.08±0.2	3.72±0.3
**Thickness**	**Pso-S**	0.0±0.0	0.0±0.0	0.8±0.3	1.80±0.5	2.65±0.3	3.10±0.2	3.55±0.2
	**Pso-W**	0.0±0.0	0.03±0.1	0.86±0.3	1.97±0.3	2.89±0.2	3.33±0.2	3.94±0.2
**PASI**	**Pso-S**	0.0±0.0	1.10±0.2	3.75±0.4	5.85±0.9	7.75±0.5	7.75±0.5	9.30±0.6
**score**	**Pso-W**	0.0±0.0	1.57±0.3	3.94±0.5	6.22±0.6	8.50±0.5	10.19±0.4	10.97±0.5

Table S2. Mean values of body weights (mean±SD)

Body weight	Ctrl-S	Ctrl-W	Pso-W-S	Pso-W-W
4 weeks	15.86±1.14	14.45±1.33	-	-
7 weeks	19.04±2.28	21.06±2.64	-	-
9 weeks	21.06±2.53	23.70±2.91	-	-
14 weeks	22.84±3.13	26.25±3.78	26.98±3.55	27.08±4.29
15 weeks	23.46±2.41	27.12±2.31	24.10±3.56	25.20±4.24
17 weeks	24.51±2.36	28.60±3.50	25.53±3.55	27.28±3.53
19 weeks	25.20±2.21	29.10±2.50	25.78±3.13	27.68±3.68
21 weeks	25.67±2.02	29.50±3.40	25.70±3.23	28.72±4.24

Table S3. Mean values of spleen weight and SW/BW ratio (mean±SD)

	Ctrl-S	Pso-S	Ctrl-W	Pso-W
**Spleen weight**	0.0818±0.013	0.1800±0.033	0.0957±0.038	0.2017±0.059
**SW/BW**	0.0037±0.001	0.0089±0.001	0.0035±0.001	0.0101±0.004

Table S4. Mean values of biochemical parameters (mean±SD)

	Ctrl-S	Pso-S	Ctrl-W	Pso-W	Pso-W-S	Pso-W-W
**Cholesterol**	159.00±4.99	154.50±3.70	183.20±13.11	180.70±7.26	152.20±3.49	189.60±8.56
**Triglycerides**	121.90±13.29	106.50±6.61	121.30±31.49	114.20±9.24	116.20±19.27	118.20±9.42

Table S5. Mean values of lymphocyte populations (mean±SD)

Cells	Ctrl-S	Pso-S	Ctrl-W	Pso-W	Pso-W-S	Pso-W-W
**CD4+**	54.90±4.60	47.01±1.85	58.91±0.87	53.69±2.79	60.02±1.51	52.18±7.98
**CD8+**	39.74±4.94	45.64±2.92	37.25±1.83	40.07±3.49	35.70±1.82	41.19±6.78
**B**	85.24±2.28	33.96±6.44	81.94±1.84	54.21±5.73	87.59±2.16	78.67±4.19
**NK**	5.13±2.02	10.42±2.47	8.27±1.36	16.90±7.90	2.40±0.41	3.18±0.82

Table S6. Mean values of circulating inflammatory cytokines/chemokines/hormones (mean±SD)

	Ctrl-S	Pso-S	Ctrl-W	Pso-W	Pso-W-S	Pso-W-W
**Eotaxin**	20.69±8.62	62.98±16.34	81.13±47.30	137.8±72.94	19.97±12.23	90.92±20.81
**ICAM-1**	67.26±29.42	471.20±64.90	275.30±142.10	1038.00±131.50	66.85±59.55	135.20±64.62
**IFN-γ**	118.70±47.15	259.80±104.50	210.80±125.80	384.70±233.80	120.90±61.54	275.00±15.09
**IL-1β**	3.21±1.80	27.95±11.56	28.40±9.47	45.00±10.98	3.23±1.57	37.30±21.74
**IL-6**	13.62±6.94	110.80±29.55	96.62±18.66	184.60±100.90	65.85±25.00	130.60±28.91
**IL-10**	5.33±3.22`	82.05±35.46	8.82±4.45	108.90±67.64	7.63±2.37	15.30±4.29
**IL-17**	8.83±2.78	47.63±10.58	23.23±10.53	51.40±12.46	18.18±9.46	32.34±10.32
**Leptin**	18.25±8.60	185.50±45.39	306.70±189.70	429.40±111.00	48.00±15.04	1517.00±440.20
**MCP-1**	88.41±25.24	297.30±54.26	248.00±125.80	534.80±240.30	186.00±30.77	331.80±132.20
**MIP-1α**	40.58±23.15	121.50±74.33	90.95±40.15	173.30±52.11	58.93±23.38	115.00±16.97
**PF-4**	3328.00±439.00	4215.00±214.70	4562.00±884.00	4802.00±90.00	3966.00±883.10	4501.00±337.60
**RANTES**	8.26±4.48	88.65±11.03	123.20±31.51	255.20±108.20	40.52±8.47	130.30±26.79
**TIMP-1**	112.60±58.24	3300.00±401.40	1908.00±987.80	4125.00±2719.00	303.90±194.70	2313.00±879.10
**TNF-α**	17.23±6.24	22.45±10.33	42.75±15.99	72.43±30.87	21.33±7.95	56.14±10.06

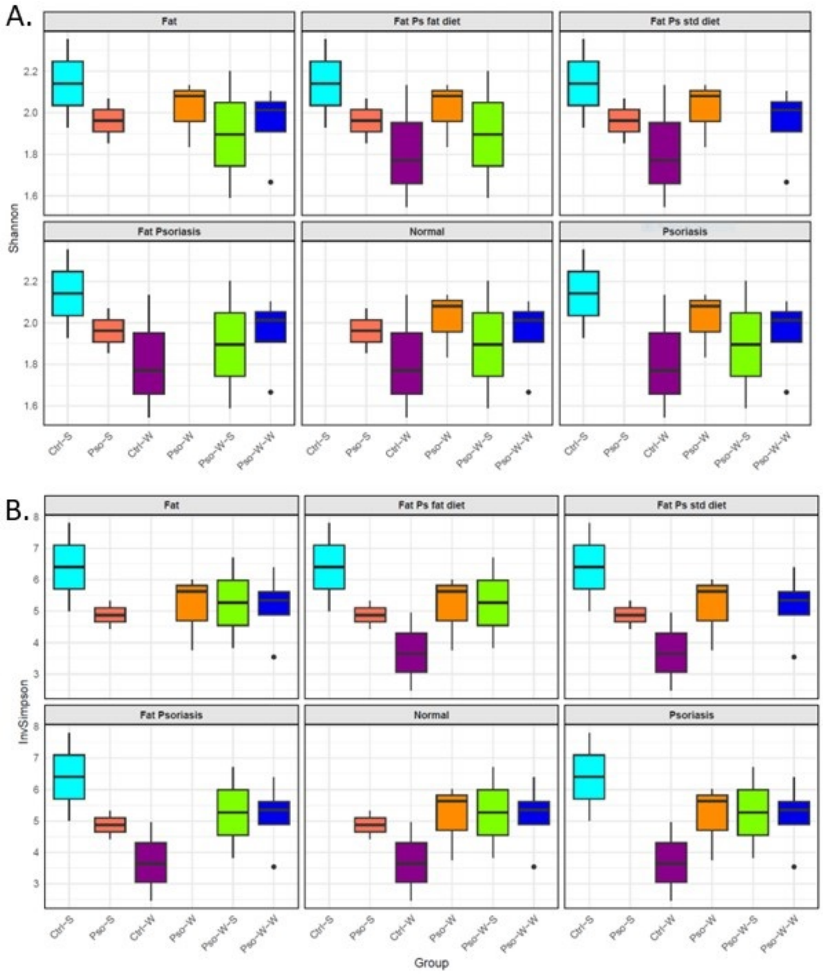

Figure S1. Evaluation of α-diversity indices (Shannon and InvSimpson) at species level across the experimental groups.

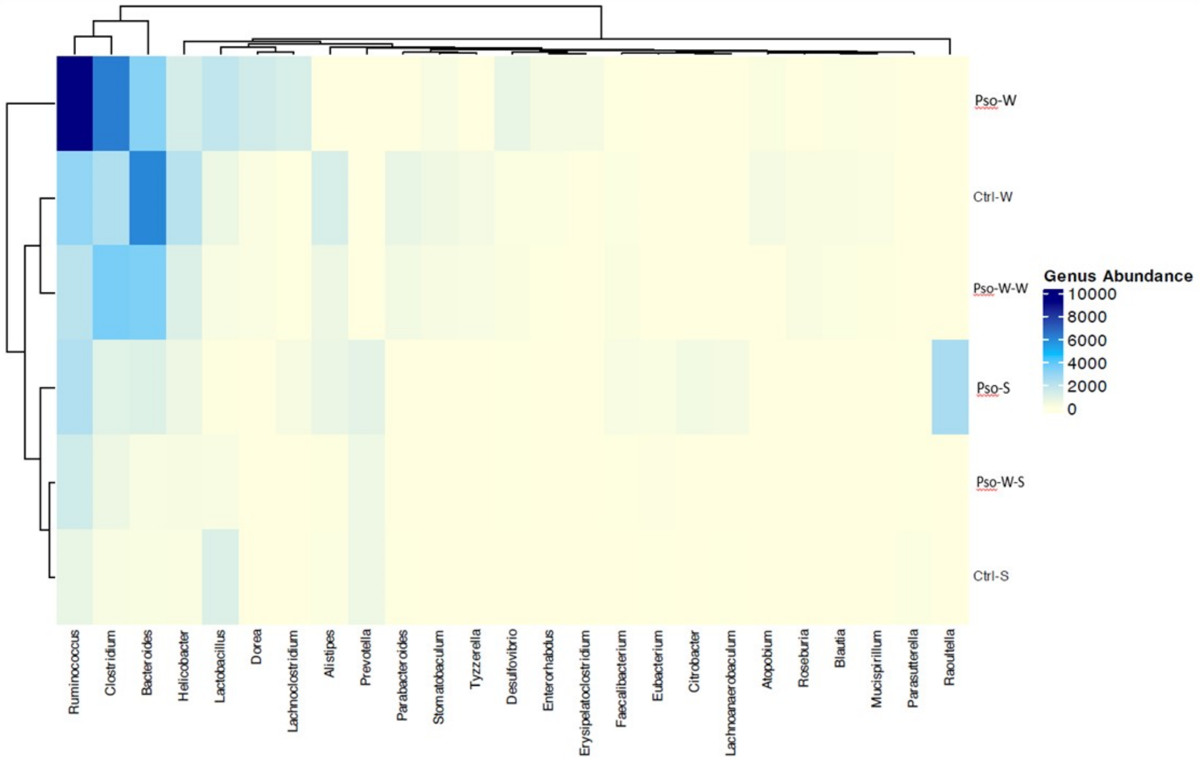

Figure S2. Heatmap comparing bacterial relative abundance across the experimental groups at the genus level.

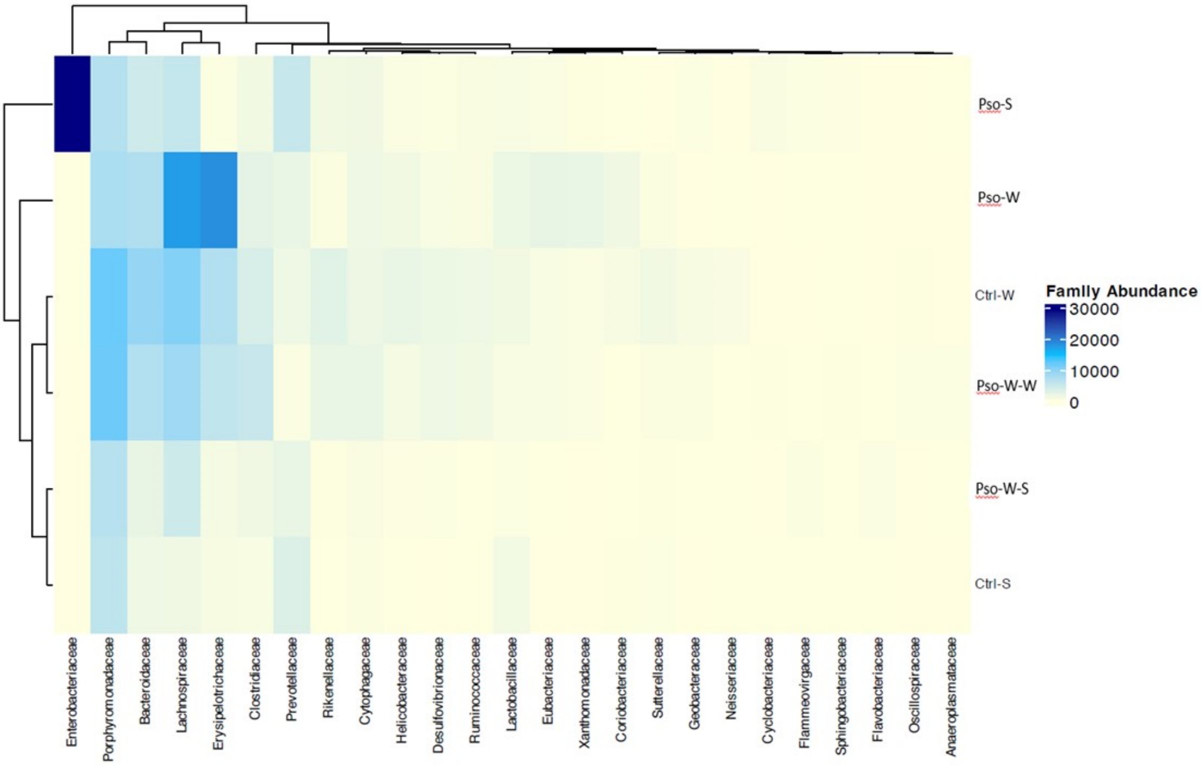

Figure S3. Heatmap comparing bacterial relative abundance across the experimental groups at the family level.

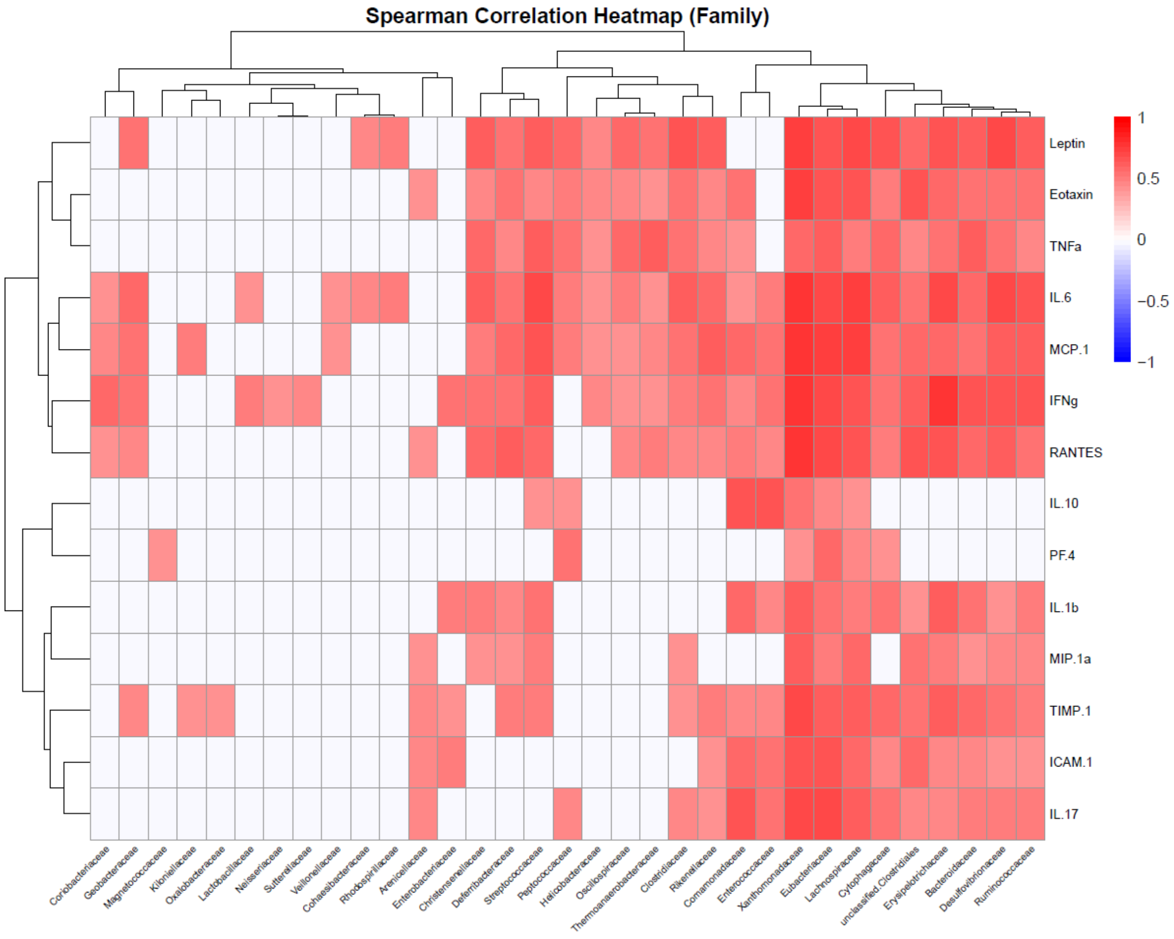

Figure S4. Heatmap of Spearman correlations between microbial families and host cytokines.

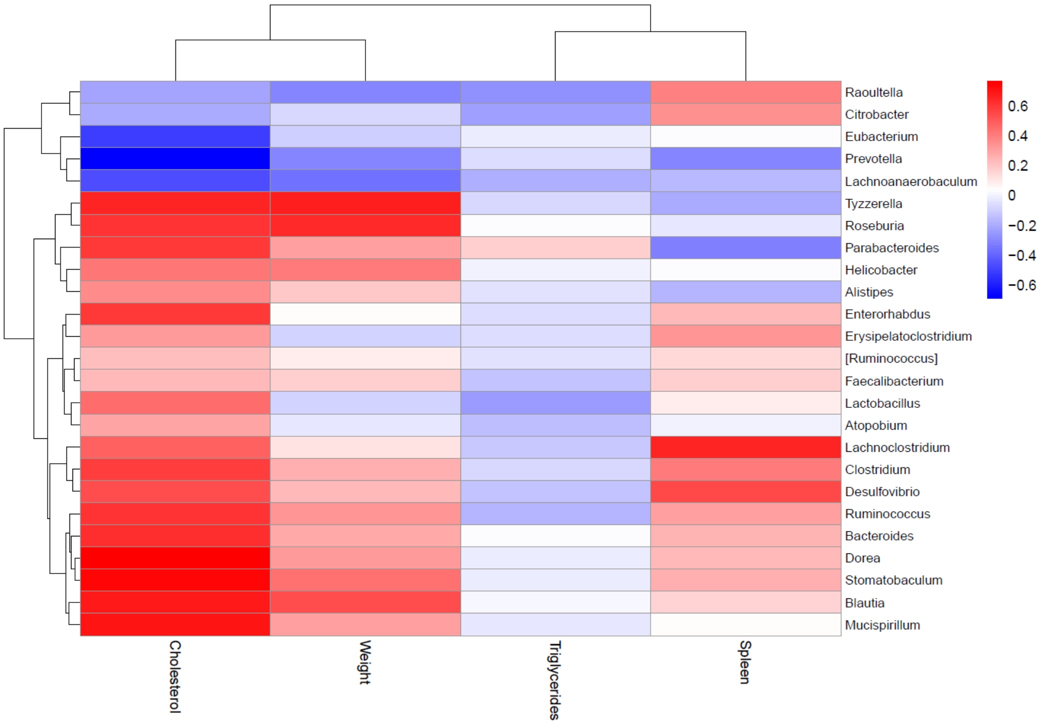

Figure S5. Correlations between relative bacterial abundance (genus level) and triglyceride levels, cholesterol levels, spleen weight and body weight.

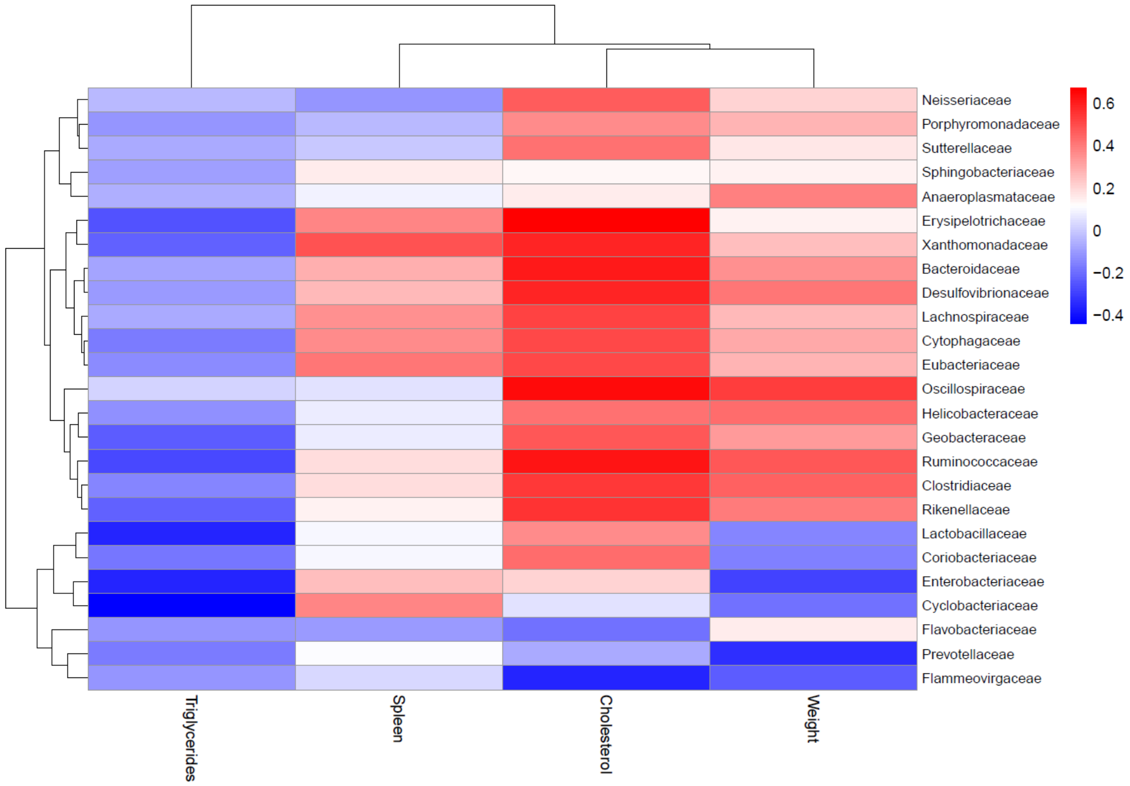

Figure S6. Correlations between relative bacterial abundance (family level) and triglyceride levels, cholesterol levels, spleen weight and body weight.
